# Culture-Dependent Bioprospecting of Halophilic Microorganisms from Portuguese Salterns

**DOI:** 10.3390/microorganisms13122867

**Published:** 2025-12-17

**Authors:** Eduarda Almeida, Adrianna Jackiewicz, Maria de Fátima Carvalho, Olga Maria Lage

**Affiliations:** 1 Faculty of Sciences, University of Porto (FCUP), Rua do Campo Alegre s/n, 4169-007 Porto, Portugalada.jackiewicz@gmail.com (A.J.); 2 CIIMAR/CIMAR LA, Interdisciplinary Centre of Marine and Environmental Research, University of Porto, Terminal de Cruzeiros do Porto de Leixões, Avenida General Norton de Matos s/n, 4450-208 Matosinhos, Portugal; mcarvalho@ciimar.up.pt; 3 ICBAS—School of Medicine and Biomedical Sciences, University of Porto, Rua de Jorge Viterbo Ferreira, 228, 4050-313 Porto, Portugal

**Keywords:** salterns, microbiota, halophile, halotolerant, polyketide synthase (PKS), non-ribosomal peptide synthetase (NRPS)

## Abstract

Extreme hypersaline environments harbour a unique biodiversity capable of surviving in such habitats, including halophilic and halotolerant bacteria. Microbial adaptations to these environments comprehend two main strategies: the “salt-in” that involves a high intracellular concentration of salts (e.g., potassium), and the “salt-out” that relies on the accumulation of small organic compounds (e.g., glycine betaine and trehalose). These evolutionary haloadaptations, combined with natural population competitiveness, often promotes the production of distinctive antimicrobial compounds, highlighting hypersaline environments as promising rich sources of novel natural products with biotechnological potential. Aiming at enlarging the knowledge on the microbiota of two Portuguese salterns (Aveiro and Olhão), microbial isolation was performed using salt and saline sediment samples. A total of 39 microbial isolates were obtained in a saline medium, affiliated with *Bacillota*, *Pseudomonadota, Actinomycetota*, and *Rhodothermaeota* and the archaeal phylum *Euryarchaeota*. All isolates are generally common in saline habitats, with most (79%) exhibiting a halotolerant profile. Regarding the presence of biosynthetic related genes, 28% of the isolates lacked type I genes for polyketide synthases or non-ribosomal peptide synthetases, 36% contained at least one of these genes, and 36% possessed both. This study provides evidence of the biotechnological potential of the microbiota from two Portuguese salterns.

## 1. Introduction

Halophiles are microorganisms found across the three domains of life, *Archaea*, *Bacteria*, and *Eukarya*, that inhabit hypersaline environments [1], such as saline lakes, salt pans, salt marshes, saline soils, or salted food products. Regarding their salt (NaCl) concentrations, they can be classified into slight (1–5%), moderate (5–20%), and extreme (20–30%) halophiles. Furthermore, there are microorganisms that can grow in both the absence or presence of high salt concentrations, which are referred to as halotolerant [2]. In order to maintain osmotic balance while living in such challenging environments, these microorganisms rely on two main survival strategies: (i) extensive molecular adaptations of their proteome to high salt concentrations, and (ii) synthesis or uptake of organic compatible solutes (also called osmolytes) [3]. In high salt concentrations, the proteomes have consistently shown a marked enrichment of small, polar, and acidic amino acids such as aspartate and glutamate, which occur mainly at the protein surface [4]. These proteins are so specialized that they interact with a solvent characterized by high ionic strength and reduced water activity, while still preserving the same functional roles as their non-halophilic counterparts. The second strategy relies on the microbial ability to exclude salts from cytoplasm along with the production and accumulation of organic-compatible solutes, such as glutamate, glutamine, proline, or ectoine, which confer osmotic balance to the microbial cells, thus preventing enzymatic inhibition, inactivation, and denaturation [5,6].

The above listed strategies clearly demonstrate the wide battery of metabolic adaptations carried out by the living microbiota inhabiting hypersaline habitats, which are also, many times, associated with other extreme environmental factors besides the high salt concentration, such as alkaline pH, high temperatures, and solar radiations [1]. These adaptations and others associated with, for example, populational competitiveness can induce the production of many natural products (NPs) with biotechnological interest. As such, halophiles have been considered good candidates for the search of novel important molecules [7,8,9].

Portugal has a coastline of 2830 km, of which 943 km are in continental territory. These 943 km harbour five major locations of salt flats, i.e., large areas with sets of several salterns, namely in Aveiro, Figueira da Foz, Tejo, Sado, and Algarve. Despite this abundance of salt pans, their microbiota is understudied. There are some reports (i) describing new strains observed in metagenomics studies from the Tavira saltern (Algarve salt flats) [10], (ii) relying on metagenomics studies from the Algarve salt flats [11,12,13], (iii) characterizing new species from the Tavira saltern [14] and from the Tejo salt flats [15], (iv) consisting of a genome announcement of an archaeal isolate from the Figueira da Foz salt flats [16], and (v) applying an extensive isolation approach targeting an Aveiro saltern and a saltern from Olhão (Algarve salt flats) without any special focus on halophiles [17,18].

For the present study, salt and sediments from two geographically distant Portuguese salterns (Aveiro and Olhão) were used, aiming at the isolation of halophiles. All strains obtained under culture were phylogenetically identified based on the 16S rRNA gene. Moreover, salinity profiles of the isolates were evaluated, as well as the presence of genes associated with the synthesis of bioactive compounds. Ultimately, the present work represents an additional contribution for enlarging the knowledge on the microbial diversity of Portuguese salterns and on its biotechnological potential, also providing different and distinctive strains for further biotechnological studies.

## 2. Materials and Methods

### 2.1. Sampling and Microbial Isolation

Wet salt and sediment from below the salt (biological crusts formed by microorganisms or other non-salt materials) samples (one from each) were collected directly from ponds of artisanal salt production at Aveiro saltern in September 2018 and Olhão in August 2018. For saltern locations see Table S1. The samples were used for microbial isolation, aiming at obtaining halophilic microorganisms. SW (seawater) medium, adapted from López-Hermoso et al. [19], was used as a NaCl-enriched medium at neutral pH and consisted of (g L^−1^) 125 NaCl, 9.75 MgCl_2_.6H_2_O, 15.25 MgSO_4_.7H_2_O, 0.25 CaCl_2_, 1.5 NaHCO_3_, 0.175 KBr, 5 yeast extract, and 16 agar.

Salt samples were dissolved to saturation and serially diluted up to 10^−3^, with both procedures using sterile seawater as solvent. Concerning sediment samples, vigorous vortexing was applied to resuspend 1 g of each saltern sediment, individually, in 1 mL of sterile seawater. From this suspension, serial dilutions up to 10^−3^ were made in sterile seawater. All samples, including the initial suspensions and their corresponding dilutions, were plated on SW agar medium and incubated at 30 °C for one month. Bacterial colonies with different morphotypes were picked and re-streaked in the same medium until axenic cultures were obtained. These cultures were then cryopreserved in 24% glycerol at −80 °C.

### 2.2. Bacterial DNA Extraction

Axenic cultures grown in SW broth were used for DNA extraction by employing the E.Z.N.A. Bacterial DNA kit (Omega Bio-Tek, Norcross, GA, USA), according to the manufacturer’s instructions. DNA quantification was carried out in the µDrop^TM^ platform (Thermo Fisher Scientific, Waltham, MA, USA) and DNA quality was screened through a 100 V electrophoresis for 30 min in a 0.8% agarose gel with 1X Tris-acetate-EDTA (TAE) buffer (Bio-Rad, Hercules, CA, USA) and stained with GreenSafe Premium (NZYTech, Lisboa, Portugal). Gels were visualized on the Bio-Rad Gel Doc XR system, Image Lab version 6.1.0 (Bio-Rad, Hercules, CA, USA).

### 2.3. Microbial Identification

The universal primers 27F and 1492R [20] were used for the PCR amplification of bacterial 16S rRNA gene, while the archaeal universal primers 340F and 1000R [21] were used to amplify the 16S rRNA gene of Archaea.

PCR reactions for bacterial DNA consisted of a mixture (25 µL) of 1 × NZYTaq 2 × Green Master Mix (NZYTech, Lisboa, Portugal), 0.1 µM of each primer, and 25 ng of DNA template. For the archaeal DNA, the only change applied to the PCR reactions preparation was the primer concentrations, where 0.5 µM of each primer was used. The PCR programme for bacterial 16S rRNA gene amplification consisted in an initial denaturation step of 5 min at 95 °C, followed by 30 cycles of 1 min at 95 °C, 1 min at 56 °C, and 1.5 min at 72 °C, and a final extension of 10 min at 72 °C. For Archaea, PCR protocol relied on an initial denaturation step of 5 min at 95 °C, followed by 30 cycles of 30 s at 95 °C, 30 s at 57 °C, and 1.5 min at 72 °C, and a final extension of 7 min at 72 °C. The MyCycler™ Thermo Cycler (Bio-Rad, Hercules, CA, USA) was used for the PCR amplification in both cases. Amplicons were visualized in the Bio-Rad Gel Doc XR system after electrophoresis in a 0.8% agarose gel with 1 × TAE buffer, stained with GreenSafe Premium. The PCR products were purified using Cytiva Life Sciences™ illustra™ GFX^TM^ PCR DNA and Gel Band Purification Kit (GE Healthcare, Buckinghamshire, UK), and amplicons were sequenced at Eurofins Genomics Germany GmbH (Ebersberg, Germany).

Sequence analyses were conducted using Geneious Prime 2023.0 (Biomatters Ltd., Auckland, New Zealand). The curated sequences were matched in the EzBioCloud [22] to their closest relatives, and the phylogenetic tree was constructed using MEGA 7 software [23]. Specifically, 16S rRNA gene sequences from both the isolates obtained in this study and their closest related strains were aligned using the ClustalW algorithm considering a Gap Open Penalty of 15.00 and a Gap Extension Penalty of 6.66. This multiple alignment was used to build a phylogenetic tree by applying the Maximum Likelihood statistical method, the phylogeny test based on Bootstrap method considering 1000 replicates, and the Tamura–Nei substitution model. The Archaea *Halobacterium salinarum* was used as outgroup.

All 16S rRNA gene sequences of the isolates were deposited in the GenBank database at the National Centre for Biotechnology Information (NCBI) database under the accession numbers MZ818003 till MZ818041.

### 2.4. Dereplication of Strains Affiliated with the Same Species

The fingerprinting methods BOX-A1R-based repetitive extragenic palindromic-PCR (BOX-PCR) and enterobacterial repetitive intergenic consensus (ERIC) PCR were applied for dereplication of the isolates affiliated with the same species. The reaction mixture for BOX-PCR consisted of 25 µL containing 1 × NZYTaq 2 × Green Master Mix, 0.3 µM primer BOXA1R (5′-CTACGGCAAGGCGACGCTGACG-3′) [24], and 25 ng DNA template. A similar PCR mixture was used for ERIC-PCR, with exception of the primers: ERIC1R (5′-ATGTAAGCTCCTGGGGATTCAC-3′) and ERIC2 (5′-AAGTAAGTGACTGGGGTGAGCG-3′) [25] were used instead of BOXA1R, each at a concentration of 0.4 µM.

The PCR protocol for BOX-PCR comprised an initial denaturation step of 7 min at 95 °C, followed by 30 cycles of 1 min at 95 °C, 1 min at 48 °C, and 8 min at 72 °C, and a final extension of 16 min at 72 °C. For ERIC-PCR, PCR programme consisted of an initial denaturation step of 7 min at 95 °C, followed by 30 cycles of 1 min at 95 °C, 1 min at 52 °C, and 8 min at 72 °C, and a final extension of 10 min at 72 °C. The MyCycler™ Thermo Cycler (Bio-Rad, Hercules, CA, USA) was used for both amplifications, and PCR products were visualized in the Bio-Rad Gel Doc XR system after electrophoresis in a 1.5% agarose gel with 1 × TAE buffer, stained with GreenSafe Premium.

### 2.5. Isolates’ Halophilic Profiles

The microbial growth of all isolates at different NaCl concentrations was assessed in SW broth medium prepared with 8 different salt concentrations, namely, 0%, 3%, 5%, 8%, 13%, 20%, 25%, and 35% (salt saturation point). The NaCl concentration used for isolation, namely, 12.5%, was used as positive control. These assays were conducted in 24-well plates, with each well containing 600 µL of broth medium with inoculum adjusted to a 0.5 McFarland units. Negative controls consisting of SW broth without inoculum (blanks) were also included in the assay. All conditions were tested in triplicate and incubated at room temperature with 200 rpm agitation for up to 2 weeks. Absorbance at 600 nm was measured to evaluate microbial growth, with the exception of the actinobacterial strain ASED46, which exhibited a non-suspension-like growth. In this case, visual evaluation of growth or non-growth was applied. End-assay aseptic controls in SW agar medium were carried out for all strains.

### 2.6. Isolates’ Bioactive Potentials

A PCR-based approach was applied for the genomic prospection of the bioactive potential of the isolates. Specifically, fragments of the β-ketosynthase (KS) domain within type I polyketide synthase genes (PKS-I) were targeted using the degenerate primers MDPQQRf (5′-RTRGAYCCNCAGCAICG-3′) and HGTGTr (5′-VGTNCCNGTGCCRTG-3′) [26]. The presence of non-ribosomal peptide synthetase (NRPS) genes was assessed using the primers MTF2 [5′-GCNGG(C/T)GG(C/T)GCNTA(C/T)GTNCC-3′, corresponding to the AGGAYVP core motif I] and MTR [5′-CCNCG(AGT)AT(TC)TTNAC(T/C)TG-3′, corresponding to the QVKIRG core motif V] [27]. For the amplification of both genes, the planctomycetal strain UC49.1 was used as positive control. PCR mixtures of 25 µL, containing 1 × NZYTaq 2 × Green Master Mix, 0.8 µM of each primer, and 25 ng DNA template were prepared. The PCR protocol consisted of an initial denaturation step of 5 min at 95 °C, 11 cycles of 1 min denaturation at 95 °C, 30 s annealing at 60 °C, and 1 min extension at 72 °C, with the annealing temperature reduced by 2 °C every cycle, followed by 30 cycles of 1 min denaturation at 95 °C, 30 s annealing at 40 °C, and 1 min extension at 72 °C, and a final extension step of 10 min at 72 °C [28]. The PCR reaction was carried out in a MyCycler™ Thermo Cycler, and the amplicons were visualized using a Bio-Rad Gel Doc XR system after electrophoresis at 80 V for 1 h in a 1.2% agarose gel prepared with 1 × TAE buffer and stained with GreenSafe Premium (NZYtech, Lisboa, Portugal). Expected sizes for NRPS and PKS amplificons are approximately 1000 and 700 bp, respectively.

## 3. Results and Discussion

**Figure 1:**
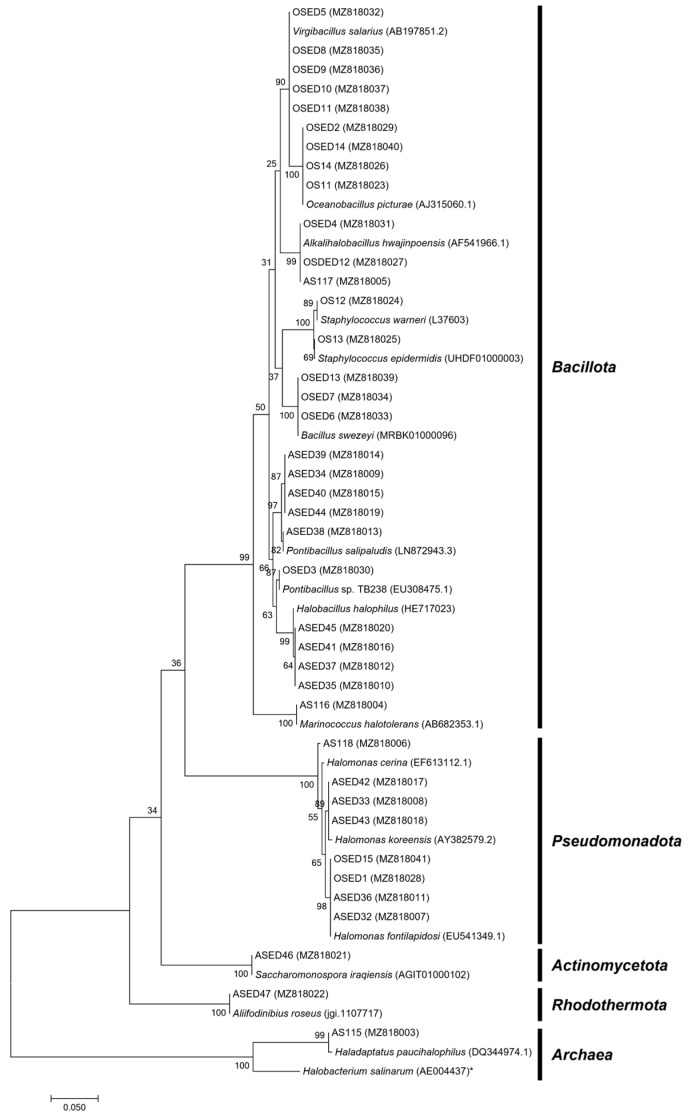
Maximum Likelihood phylogenetic tree based on 16S rRNA gene sequences showing the relationship between saltern isolates and their closely related described strains. The tree was constructed using the MEGA7 [23], with confidence assessed by bootstrapping values of 1000 replicates. * The archaeal species *Halobacterium salinarum* (AE004437) was used as out-group. Bar, 0.050 substitutions per nucleotide position.

**Figure 2:**
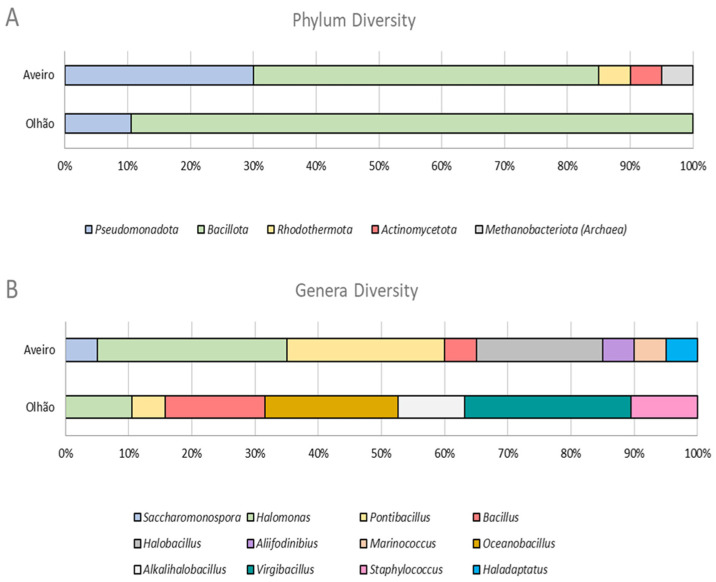
Microbial diversity at phylum (**A**) and genus (**B**) levels obtained from salt and sediment samples from two Portuguese salterns, Aveiro and Olhão.

The culture-dependent methodology applied allowed for the isolation of 39 microorganisms, 20 from Aveiro and 19 from Olhão. These microorganisms were phylogenetically distributed across two domains of life and five phyla, as shown in the phylogenetic tree presented in Figure 1. The *Archaea* domain is represented by a single *Halobacteriota* isolate with 99.65% 16S rRNA gene sequence similarity to *Haladaptatus paucihalophilus*. Most of the microorganisms obtained belong to the *Bacteria* domain and were distributed across four phyla, with the vast majority being phylogenetically related to *Bacillota* (71.7%, n = 28). Members of *Pseudomonadota* represented 20.5% (n = 8) of the cultured fraction, while the phyla *Actinomycetota* and *Rhodothermaeota* only represented 2.6% (n = 1) each. Regarding location, phylum-level diversity in Olhão was lower, with only two different phyla identified, *Pseudomonadota* (10.5%, n = 2) and *Bacillota* (89.5%, n = 17). In contrast, in Aveiro, five phyla were recovered, specifically *Pseudomonadota* (30%, n = 6), *Bacillota* (55%, n = 11), *Rhodothermaeota* (5%, n = 1), *Actinomycetota* (5%, n = 1), and *Euryarchaeota* (*Archaea*; 5%, n = 1) (Figure 2A). A quite similar phyla profile to Aveiro was observed in Romanian saline lakes [29]. Despite the low phyla diversity observed in Olhão isolates, the genus abundance was quite similar in both locations, since eight different genera were obtained from Aveiro and seven from Olhão (Figure 2B). The different genera recovered in this study have already been associated with saltern extreme hypersaline environments.

**Figure 3:**
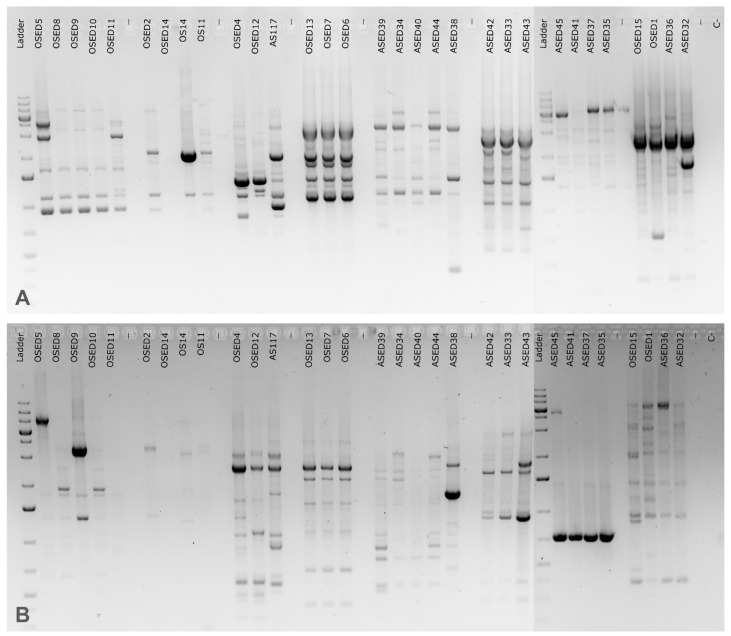
Results of the dereplication of saltern isolates affiliated with the same species through the fingerprinting methods BOX-PCR (**A**) and ERIC-PCR (**B**).

In Table 1, the halophilic profiles are provided. Figure 3 shows the fingerprinting profiles that are discriminated in Table 1. Further details on the isolates, like the affiliation similarity, are given in Table S1.

Having a look more in detail, the archaeal isolate AS115 from Aveiro showed a 99.65% 16S rRNA gene sequence similarity to the halophilic strain *Haladaptatus paucihalophilus*, which was isolated from the Zodletone Spring NSF Microbial Observatory in Oklahoma (USA) and exhibits optimal growth at 18% NaCl [30]. Isolate AS115 confirmed its halophilic nature (growth from 8% to 20% NaCl). In addition, strains closely related to AS115 and *H. paucihalophilus* have also been identified in other saline environments in India and Brazil (NCBI accession numbers JX428947 and KF650688, and KC313060, respectively), as well as in coastal environments in Essex, UK [31].

The *Rhodothermaeota* isolate ASED47 showed close phylogenetic affiliation with *Aliifodinibius roseus*, originally isolated from a salt mine [32]. To the best of our knowledge, this species was never associated with salterns before. Concerning *A. roseus*, Wang et al. [32] reported growth under NaCl concentrations from 4 to 20%, but the halophilic profile of ASED47 was not determined, because this isolate lost viability after cryopreservation.

The *Actinomycetota* strain ASED46 showed a phylogenetically close relationship with *Saccharomonospora iraqiensis*. This described species was obtained from extreme saline soils in Iraq [33] and is also reported to occur in Sahara soils (NCBI accession number FJ968730) and at a salty beach on the Tarim Basin river, China (NCBI accession number JX244088). Strain ASED46 showed to be halophilic (growth from 3 to 13% NaCl), while *S. iraqiensis* IQ-H1^T^, previously known as *Actinopolyspora iraqiensis*, showed growth from 5 to 20% NaCl [33].

Of the eight *Pseudomonadota* obtained, four (OSED1, OSED15, ASED32, and ASED36) were closely affiliated with *Halomonas fontilapidosi*, three (ASED33, ASED42, and ASED43) showed a phylogenetic affiliation with *Halomonas koreensis*, and one (AS118) was affiliated with *Halomonas cerina*. Members of the *Halomonas* genus were isolated from a wide range of saline habitats [1,34,35,36,37,38,39], and the referred species have already been recovered from the Olhão saltern [17].

Isolates OSED1, ASED32, and ASED36 proved to be halophilic (growth from 3 to 20% NaCl), while isolate OSED15 exhibited a halotolerant profile. *H. fontilapidosi* was isolated from an endorreic saline wetland in Spain and is reported to be halophilic [40]. Although these four isolates were affiliated with the same species, fingerprinting analyses revealed that each represents a distinct strain of *H. fontilapidosi*. Regarding isolates ASED33, ASED42, and ASED43, only isolate ASED42 exhibited a halophilic profile comparable to the previously described *H. koreensis*, isolated from a solar saltern in Korea [41]. Additionally, BOX- and ERIC-PCRs showed that only two of these three isolates (ASED33 and ASED42) are representatives of the strain *H. koreensis*. Isolate AS118 showed 97.9% 16S rRNA gene sequence similarity to its closest known relative *Halomonas cerina*. This described species was specifically isolated from the saline environment of Rambla Salada in Spain [36,42]. As a threshold of 99% can be considered to distinguishable between different species [43], AS118 probably represents a new species within the *Halomonas* genus. Isolate AS118 was shown to possess a halophilic profile similar to *H. cerina* [42].

Concerning *Bacillota*, eight genera in a total of 28 strains were isolated. These include members of *Alkalihalobacillus* (n = 3), *Bacillus* (n = 3), *Halobacillus* (n = 4), *Oceanobacillus* (n = 4), *Pontibacillus* (n = 6), *Virgibacillus* (n = 5), *Marinococcus* (n = 1), and *Staphylococcus* (n = 2).

Isolates AS117, OSED4, and OSED12 were phylogenetically close to *Alkalihalobacillus hwajinpoensis*, but they represent distinct strains within this species. *A. hwajinpoensis* has been associated before with the bacterial diversity of Aveiro and Olhão salterns [17], as well as with worldwide salterns [42,44]. These three strains showed a halotolerant profile (growth from 0% to 13% or 20% NaCl). *A. hwajinpoensis* (previously known as *Bacillus hwajinpoensis*) was characterized as a halophile, being able to grow at NaCl concentrations between 0.1 and 19% [45].

The three strains phylogenetically affiliated with *Bacillus swezeyi* (OSED6, OSED7, and OSED13) were shown to be halotolerants (growth from 0% up to 13% NaCl) and represented the clonal line. *B. swezeyi* was isolated from a soil sample (1–3 cm depth) collected from Evolution Canyon III at Nahal Shaharut in the southern Negev Desert in Israel, demonstrating its ability to adapt to extreme conditions, with growth from 0% to 12% NaCl [46].

Of the four isolates affiliated with *Halobacillus halophilus* (ASED35, ASED37, ASED41, and ASED45), three of them lost viability after cryopreservation. Therefore, the halophilic profile was only assessed for ASED45 that grew from 0% to NaCl saturation (<35% NaCl). *H. halophilus*, first described in 1983 under the name *Sporosarcina halophila* and isolated from salt marsh soils, could grow from 0 to 15% NaCl [47]. Related strains have also been reported in the salterns of Santa Pola, Spain [48]. Fingerprinting studies also showed that two out of the four *H. halophilus* isolates (ASED35 and ASED37) are colonal.

The four isolates affiliated with *Oceanobacillus picturae* (OS11, OS12, OSED2, and OSED14) represent distinct strains. A similar prevalence was previously reported in Olhão salterns but not in Aveiro and other salterns [49,50]. All *O. picturae*-related isolates grew at NaCl concentrations ranging from 0% to 13% or 20%. The description of *O. picturae* (formerly known as *Virgibacillus picturae*) was based on eight different isolates, all of which were capable of growing at NaCl concentrations between 0% and 10% [51].

The five isolates (ASED34, ASED38, ASED39, ASED40, and ASED44) affiliated with *Pontibacillus salipaludis* (originally described from an Indian saltern; [52]) represent distinct strains within this species. The halophilic profile could only be assessed for ASED34 and ASED39 (growth from 0% to 25% NaCl) and ASED44 (growth between 0% and 8% NaCl), indicating different NaCl requirements compared to the described *P. salipaludis* strain, which showed growth at 1 to 19% NaCl [53]. Another *Pontibacillus* isolate (OSED3) was affiliated with an undescribed *Pontibacillus* species previously isolated from a Greek solar saltern [49]. The second most closely related strain to OSED3 was *Pontibacillus marinus*, with 96.49% similarity, which was isolated from a dry salt lake in China [39]. Moreover, OSED3 showed a halotolerant profile by growing at 0 to 13% NaCl.

Although *Virgibacillus salarius* has been described to be unable to grow in the absence of salt [52], all five isolates obtained in our study were able to grow from 0 to 13% NaCl. *V. salarius* was obtained from dry salt lakes in the Tunisian Sahara and was also reported in salterns from Bulgaria [37]. Of the five isolates, OSED8 and OSED10 may represent the same strain.

The isolate AS116 related to the species *Marinococcus halotolerans*, showing the same halophilic profile as the *M. halotolerans* type of strain [54], with both being able to grow from 0 to 25% NaCl. The prevalence of the strains of *M. halotolerans* in salterns has been widely reported [48,49].

In general, all the isolates obtained were affiliated with strains associated with saline environments and showed optimum growth at NaCl concentrations similar to those described for their phylogenetically close type strains. The exception was the two *Staphylococcus* isolates (OS12 and OS13, namely, *S. warneri* and *S. epidermidis*, respectively), which are human skin commensals [55], but also are described in many other environments. Their presence in salt samples from the Olhão saltern might be a consequence of human activity in this specific site, as in addition to artisanal salt production, saltern baths are also a common tourist activity there.

**Figure 4:**
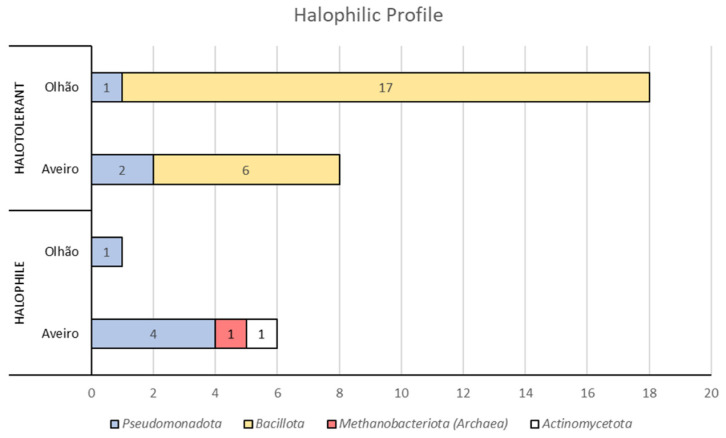
Halophilic profile, at phylum level, of the isolates obtained from salt and sediment samples collected at Aveiro and Olhão salterns.

Overall, the halophilic profile of the isolates discussed above, taking into account their phyla taxonomic rank, is summarized in Figure 4.

The biotechnological potential of all isolates obtained in this study was screened through a PCR method targeting PKS-I and NRPS genes (Table 2 and Table S1). The microbial ability to produce metabolites with biotechnological applications is not exclusively linked to PKS and NRPS genes, as many other genes can also be responsible for the production of bioactive NPs [56]. Nonetheless, Zothanpuia et al. [57] demonstrated that the presence of these genes in microorganisms are good indicators for the production of bioactive compounds. Moreover, many NPs, namely, polyketides (PK), non-ribosomal peptides (NRP), or even PK/NRP hybrids, represent compounds with economical and pharmacological relevance [58,59]. Of the 39 microorganisms analyzed, 11 (28%) did not exhibit any potential PKS or NRPS genes, while 28 (72%) potentially carried either one or both types of genes: 6 isolates (15%) were PKS-positive, 8 isolates (21%) were NRPS-positive, and 14 isolates (36%) contained both gene types.

Members of the genus *Halomonas* are recognized for their biotechnological potential associated with PKS and NRPS genes [60,61,62], as well as for their ability to produce exopolysaccharides [63,64,65,66], synthetize compatible solutes [67], and degrade aromatic compounds [68]. Furthermore, PKS and NRPS biosynthetic gene clusters have been widely documented in *Bacillus* members [69,70,71], underpinning their ability to produce a diverse array of NPs. Such biosynthetic potential enhances *Bacillus* fitness in competitive environments, exemplified by pharmacologically important antibiotics like bacillaene and bacitracin [71].

## 4. Conclusions

The Olhão saltern isolates obtained in this study were predominantly halotolerant, since only 1 isolate out of 19 affiliated with *H. fontilapidosi*, showed a strict requirement of NaCl. A more balanced population of isolates was obtained from the Aveiro saltern, with 43% of isolates presenting a halophilic profile and 57% a halotolerant profile. Specifically, all the *Bacillota* isolates were halotolerant. Moreover, although many isolates were affiliated with the same species, fingerprinting using BOX- and ERIC-PCR revealed a low prevalence of identical strain representatives, indicating the presence of intraspecies diversity. Additionally, with a few exceptions, the two fingerprinting methods applied provided a good overlap of results.

The high demand in the discovery of novel NPs with pharmaceutical applications as antimicrobial and anticancer compounds led to the search of unexplored environments like hypersaline environments. The efforts carried out by the scientific community in recent decades recognized hypersaline habitats as rich pools of undiscovered organisms with distinctive biotechnological potential besides pharmaceutical [72,73]. The saltern isolates obtained in this study represent an important source of microorganisms for further research works aiming at finding novel bioactive NPs. This is further supported by the high proportion of isolates (72%) harbouring bioactivity-related genes. Furthermore, saline microorganisms are well known for their potential regarding hydrolytic enzymes [74] but scarcely known for their bioactive potential regarding NPs. Our results also enlighten their potential for the biotechnological production of NPs. For example, the bioactive properties of haloarchaea remain understudied when compared to other major groups as bacteria, plants, or algae. However, the archaeal isolate AS115 obtained in this study harboured an NRPS gene in its genome.

The results described here confirm previous findings from the same two salterns, where a seawater-level saline medium was employed [18]. Specifically, *H. fontilapidosi*, *A. hwajinpoensis*, *O. picturae*, and *S. warneri* isolates were transversal to both studies, indicating them as putatively autochthonous strains of these salterns.

## Figures and Tables

**Table 1:** Isolates obtained in this study, and respective data on the samples’ designation, phylogeny, halophilic profile (NaCl range), and fingerprinting profiles. Each different gel band pattern is associated with a different letter: lowercase for BOX-PCR and uppercase for ERIC-PCR.

Isolate	Phylum	Genus	% NaCl Range	BOX-PCR Pattern ^a^	ERIC-PCR Pattern ^a^
AS115	*Euryarchaeota (Archaea)*	*Haladaptatus*	8 to 25	ND	ND
ASED46	*Actinomycetota*	*Saccharomonospora*	3 to 13	ND	ND
OSED4	*Bacillota*	*Alkalihalobacillus*	0 to 13	g	G
OSED12	*Bacillota*	*Alkalihalobacillus*	0 to 20	h	H
AS117	*Bacillota*	*Alkalihalobacillus*	0 to 20	i	I
OSED13	*Bacillota*	*Bacillus*	0 to 13	j	J
OSED6	*Bacillota*	*Bacillus*	0 to 13	j	J
OSED7	*Bacillota*	*Bacillus*	0 to 13	j	J
ASED35	*Bacillota*	*Halobacillus*	ND	s	S
ASED37	*Bacillota*	*Halobacillus*	ND	s	S
ASED45	*Bacillota*	*Halobacillus*	0 to 35	s	α
ASED41	*Bacillota*	*Halobacillus*	ND	t	S
AS116	*Bacillota*	*Marinococcus*	0 to 25	ND	ND
OS11	*Bacillota*	*Oceanobacillus*	0 to 13	d	D
OSED2	*Bacillota*	*Oceanobacillus*	0 to 13	d	Y
OSED14	*Bacillota*	*Oceanobacillus*	0 to 13	e	E
OS14	*Bacillota*	*Oceanobacillus*	0 to 20	f	F
ASED39	*Bacillota*	*Pontibacillus*	0 to 25	k	K
ASED34	*Bacillota*	*Pontibacillus*	0 to 25	l	L
ASED40	*Bacillota*	*Pontibacillus*	ND	m	M
ASED44	*Bacillota*	*Pontibacillus*	0 to 8	n	N
ASED38	*Bacillota*	*Pontibacillus*	ND	o	O
OSED3	*Bacillota*	*Pontibacillus*	0 to 13	ND	ND
OS13	*Bacillota*	*Staphylococcus*	0 to 8	ND	ND
OS12	*Bacillota*	*Staphylococcus*	0 to 13	ND	ND
OSED5	*Bacillota*	*Virgibacillus*	0 to 13	a	A
OSED10	*Bacillota*	*Virgibacillus*	0 to 13	b	B
OSED8	*Bacillota*	*Virgibacillus*	0 to 13	b	B
OSED9	*Bacillota*	*Virgibacillus*	0 to 13	b	Z
OSED11	*Bacillota*	*Virgibacillus*	0 to 13	c	C
ASED42	*Pseudomonadota*	*Halomonas*	3 to 20	p	P
ASED33	*Pseudomonadota*	*Halomonas*	0 to 20	p	P
ASED43	*Pseudomonadota*	*Halomonas*	0 to 20	q	Q
OSED15	*Pseudomonadota*	*Halomonas*	0 to 20	u	U
OSED1	*Pseudomonadota*	*Halomonas*	3 to 20	v	V
ASED36	*Pseudomonadota*	*Halomonas*	3 to 20	w	W
ASED32	*Pseudomonadota*	*Halomonas*	3 to 20	x	X
AS118	*Pseudomonadota*	*Halomonas*	3 to 20	ND	ND
ASED47	*Rhodothermaeota*	*Aliifodinibius*	ND	ND	ND

**Table 2:** Bioactive potential of isolated genera from Aveiro and Olhão, represented through the number of isolates harbouring potential PKS-I, NRPS, or both genes.

Phylum	Genus	Total Number of Isolates	Only PKS-I	Only NRPS	Both
*Actinomycetota*	*Saccharomonospora*	1	0	0	0
*Pseudomonadota*	*Halomonas*	8	1	2	1
*Bacillota*	*Pontibacillus*	6	2	0	2
	*Halobacillus*	4	0	2	2
	*Marinococcus*	1	0	0	1
	*Bacillus*	3	1	0	2
	*Oceanobacillus*	4	0	0	2
	*Alkalihalobacillus*	3	1	0	2
	*Virgibacillus*	5	1	1	2
	*Staphylococcus*	2	0	1	0
*Rhodothermaeota*	*Aliifodinibius*	1	0	0	1
*Euryarchaeota (Archaea)*	*Haladaptatus*	1	0	1	0

## Data Availability

Repositories: Sequences data were deposited at GenBank database under accession numbers from MZ818003 to MZ818041.

## Supplementary Materials

The following supporting information can be downloaded at: https://www.mdpi.com/article/10.3390/microorganisms13122867/s1, Table S1: Information on the isolates obtained from Aveiro and Olhão salterns.

## Abbreviations

The following abbreviations are used in this manuscript:

NRPS	Non-ribosomal Peptide Synthetase
PKS	Polyketide Synthase
NPs	Natural Products
ERIC	Enterobacterial Repetitive Intergenic Consensus
BOX	Box Repetitive Extragenic Palindromic
PCR	Polymerase Chain Reaction
KS	β-ketosynthase
